# Development and characterization of aceclofenac–LDH/alginate porous microspheres for pH-responsive drug release

**DOI:** 10.1039/d6ra03347d

**Published:** 2026-08-12

**Authors:** Yanling Chen, Xiaowen Wu

**Affiliations:** a School of Resources and Environment Engineering, Shandong Agriculture and Engineering University Jinan 250100 China wuxiaowen1114@163.com

## Abstract

The antipyretic and anti-inflammatory drug aceclofenac (ACF) causes the serious side effect of adverse gastrointestinal reactions. With the aim to provide sustained action and prevent drug release into the stomach, aceclofenac–LDH/alginate porous microspheres were developed *via* an emulsion method to achieve sustained and pH-responsive drug release. The obtained microspheres were characterized by XRD, SEM, FT-IR, and TG–DSC. The SEM images show a porous morphology of microspheres with diameters of 2 µm. TG–DSC data prove that the thermal stability was greatly increased by the interaction of the LDH and alginate. *In vitro* drug release was studied in PBS (pH 7.4 and 4.0) at 37 °C. Compared with the physical mixture and ACF–LDH nanohybrids, ACF release from the porous LDH/Alg microspheres was 50% (pH 4.0) within 16 h, whereas 62% of the drug was released at pH 7.4, indicative of a sustained and pH-responsive drug release profile.

## Abstract 2

Porous LDH/alginate microspheres were designed for controlled release of aceclofenac, which performed sustained and pH-responsive drug release profiles. The microspheres were characterized by XRD, SEM, FT-IR, and TG–DSC techniques.

## Graphical abstract

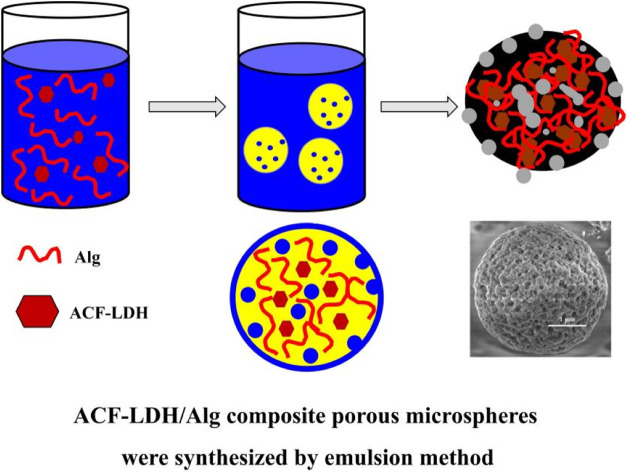

## Introduction

As the most convenient route for clinical therapy, oral administration is applied to many kinds of drugs.^1,2^ For non-steroidal anti-inflammatory drugs (NSAID) in particular, oral delivery has high patient compliance and is the most preferable route for long-term treatment.^3^ However, traditional oral administration has disadvantages, such as gastrointestinal damage, low bioavailability, and the need for frequent and high doses.^4^ Aceclofenac (ACF), a new kind of NSAID which is efficient for the treatment of osteoarthritis and rheumatoid arthritis, is limited by its poor solubility in water, short half-life and the adverse gastrointestinal reaction it causes during clinical application.^5,6^ To decrease the dosing frequency and adverse effects in the gastrointestinal tract, a sustained and pH-responsive drug release system are considered to be the proper approach. The development of novel oral delivery systems is necessary to overcome these shortcomings and increase the convenience of oral medication.^3,7^ The choice of a suitable drug delivery system is important to control drug release.

During the past few decades, numerous organic or inorganic materials have been investigated for application in drug delivery systems,^8^ including inorganic nanoparticles,^9^ natural or synthetic polymers,^10–13^ polymer–lipid hybrid nanoparticles^14^ and organic–inorganic nanocomposites.^15^ In particular, layered double hydroxides (LDHs) have attracted much interest and have been widely used in various biomedical applications. Layered double hydroxides are well-known as biocompatible anion clays,^9,16,17^ which can be represented by the general formula [M_1−*x*_^2+^M_*x*_^3+^(OH)_2_](A^*n*−^)_*x*/*n*_·*m*H_2_O, where M^2+^ and M^3+^ are di- and trivalent cations, respectively, and A^*n*−^ is the interlayer anion. Due to isomorphous substitution, the layers of LDHs possess positive charges which are balanced by interlayer hydrated anions and water molecules. Different kinds of biological molecules or drug anions can be intercalated into the interlayers of LDHs to form nanohybrids.^9,16^ Due to their low toxicity, low rates of biodegradation and ability to neutralize gastric acid, various nanohybrids can be utilized as drug carriers and their drug release can be controlled by host–guest interactions.^16–18^ Unfortunately, the oral clinical application of hydrotalcite-like compounds is limited by their tendency to rapid release in the acid stomach environment, their low bioavailability in the small intestine and need for frequent administration.^19^ The development of LDH-based drug delivery systems is required to improve clinical efficacy.^17^ Recently, LDH-polymer composites have been investigated as drug delivery systems due to their multi-stimuli responsiveness and biocompatibility. The LDH core dissolves in response to the acidic microenvironment (pH-sensitive), releasing the intercalated drug. Simultaneously, the polymer shell can be engineered to respond to other triggers. The composites often allow for a two-stage release profile: initial protection by the polymer, followed by release from the LDH, resulting in controlled drug release.^20^

Sodium alginate is attractive due to its low cost, biocompatibility, biodegradability, non-toxicity and mild gelling conditions.^21^ However, the application of sodium alginate in drug delivery systems is limited by the instability and its poor mechanical characteristics.^22^ Different kinds of inorganic materials such as calcium carbonate, hydroxyapatite and graphene oxide have been added to sodium alginate to form composites which combine the properties of an elastic organic polymer and inorganic nanoparticles.^23–25^ The composites possess good stability, mechanical properties and porosity.

In this study, LDH materials were formulated into polymers to form composites with the aim of facilitating the sustained and pH-responsive release of aceclofenac. We have designed porous LDH–alginate nanocomposites (LDH/Alg) *via* an emulsion method, which can prolong the half-life, reduce administration time and decrease gastrointestinal tract side effects. The ACF intercalated LDH nanohybrids were synthesized *via* an exfoliation–co-assembly route. The ACF molecules were successfully intercalated into the interlayers of the LDH and their release can be controlled using host–guest interactions. To achieve controlled release, the ACF–LDH nanohybrids were interacted with calcium alginate to prepare porous microspheres *via* an emulsion method. This study for the first time reports the design of ACF-containing LDH/Alg porous microspheres for achieving sustained and pH-responsive drug release. As an anti-gastric acid drug, LDH and alginate can improve the drug delivery and effectively weaken the response of aceclofenac to the gastrointestinal tract, while the composites can control the release of ACF in the acidic stomach environment.

## Experimental

### Reagents

**Fig. 1:**
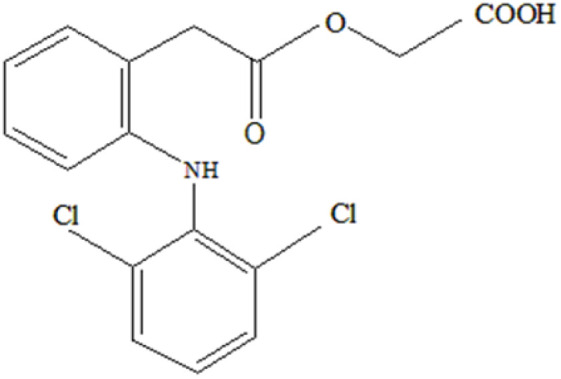
Molecular structure of aceclofenac.

Chemicals in this work were used as obtained: sodium alginate (average *M*_n_ = 354) was purchased from Sigma-Aldrich. Aceclofenac (99% purity) was purchased from the Wuhan Yuancheng Technology Co., Ltd (China) and used as received. The molecular structure of aceclofenac is shown in Fig. 1. Mg(NO_3_)_2_·6H_2_O and Al(NO_3_)_3_·12H_2_O were purchased from Tianjin Shengao Chemical Reagent Co., Ltd (China). Calcium chloride and dimethyl sulfoxide were purchased from Tianjin Zhiyuan Chemical Reagent Co., Ltd (China). Span 80 was purchased from Shanghai Maclean Biochemical Technology Co., Ltd (China). Ammonium hydroxide was purchased from Sichuan Xilong Chemical Co., Ltd (China). Phosphate buffer saline was purchased from Boster Biological Technology Co., Ltd (China). Formamide was purchased from China National Pharmaceutical Group Corporation. The reagents used were of analytical grade. The experimental water was processed in the laboratory.

### Synthesis of ACF–LDHs

A pristine Mg_3_Al–NO_3_ LDH sample was synthesized according to a coprecipitation method.^26^ The delamination of the LDH sample was performed according to the method described by Wu *et al.*^27^ Briefly, an LDH sample (2.0 g) was dispersed in 100 mL of formamide under magnetic stirring, and the resulting dispersion was sonicated until it became transparent to provide a 20 g L^−1^ formamide dispersion of LDH nanosheets.

ACF–LDH nanohybrids were prepared using a co-assembly route.^28^ First, 10 g of aceclofenac was dissolved in 25 mL of DMSO. A 2.5 mL portion of the 20 g L^−1^ LDH nanosheet dispersion was added to the ACF–DMSO solution. Following a 0.5 h period of standing at room temperature, the suspension was centrifuged and washed sequentially with deionized water and anhydrous ethanol using a re-dispersion/centrifugation process. The resulting solid sample, denoted as ACF–LDH, was dried at 60 °C in a vacuum oven.

### Synthesis of ACF–LDH/Alg microspheres

**Fig. 2:**
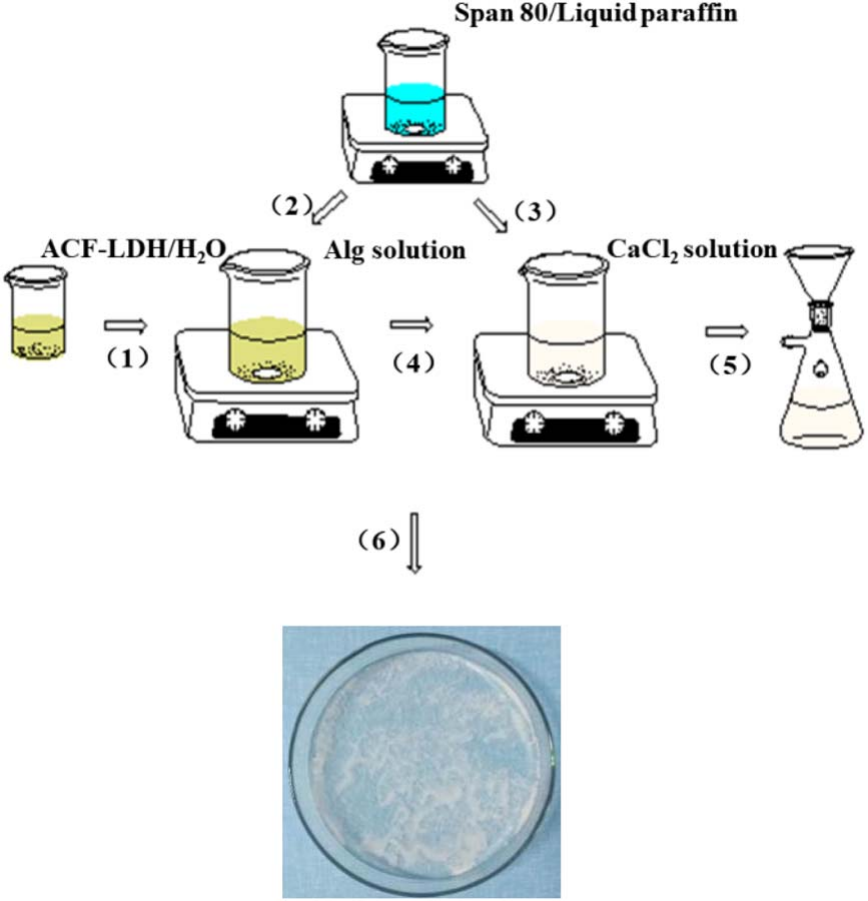
Schematic illustration of the processes used for preparing the ACF–LDH/Alg composite microspheres.

The ACF–LDH/Alg composite microspheres were prepared in a water-in-oil (W/O) emulsion system, as shown in Fig. 2. Firstly, 0.8 g of the ACF–LDH nanohybrids was dispersed in 10 mL of distilled water with sonication for 5 min, which was added into 10.0 mL of a sodium alginate solution (wt = 4%) with stirring at 40 °C for 30 min. Then, 1.78 g of Span 80 was dissolved in 210 mL of liquid paraffin at 40 °C. At 40 °C, 60 mL of the Span 80/paraffin solution was added to the ACF–LDH/SA dispersion, which was stirred at 40 °C for 60 min to form a W/O emulsion. Secondly, 150 mL of the Span 80/paraffin solution was added into 50 mL of a CaCl_2_ aqueous solution (wt = 5%) and the system was stirred at 40 °C for 60 min. Finally, these two emulsions were blended together with vigorous mechanical mixing at 40 °C for 1 h. After standing for 24 h, the obtained white precipitation was collected by filtration and washed sequentially with petroleum ether and ethanol. The resulting solid sample, denoted as ACF–LDH/Alg microspheres, was dried at 40 °C in a vacuum oven.

### X-ray crystallography

X-ray diffraction (XRD) patterns were recorded with a Bruker-D8 advanced diffractometer equipped with Cu Kα radiation (*λ* = 1.54056 Å) at 40 kV and 40 mA.

### Infrared spectroscopy

IR spectra were measured with a Shimadzu IRAffinity-1 Fourier transform infrared spectrometer in the range from 400 to 4000 cm^−1^ in air at ambient temperature using the KBr disk technique.

### FE-SEM

The sample morphology was analyzed using a JSM-7610FPlus scanning electron microscopy with a 1 kV acceleration voltage and gold spraying.

### TEM

Transmission electron microscopy (TEM) images were observed with a JEM-2100 model transmission electron microscope.

### TG–DSC

Thermal characterization of aceclofenac, the pristine LDH, ACF–LDH, ACF–Alg microspheres and ACF–LDH/Alg microspheres was conducted using a thermogravimetric analyzer (TGA/SDTA851). The thermograms were collected using a heating ramp of 10 °C min^−1^ in a temperature range of 30–1000 °C.

### Examination of the release rate

The *in vitro* release of ACF was examined at 37 °C in phosphate buffer solution (PBS, pH 7.4, pH 4.0, 0.02 M). The nanohybrid or microspheres (50 mg) were added into the PBS solution (1000 mL), which was stirred at 37 °C. Aliquots (3 mL) of the suspension were then withdrawn at predetermined time intervals and immediately filtered through a 0.45 µm membrane filter. The concentrations of ACF in the filtrates were measured using a UV-vis spectrometer at *λ* = 275 nm to calculate the release amounts (*q*_*t*_) and the percentage release values (*X*_*t*_) of the ACF from the nanohybrid. All of these tests were performed in triplicate and the final values reported as an average of these measurements. The percent release values of the ACF were plotted *versus* time (*t*) to examine the release rate of the drug from the nanohybrid. To verify the release process of ACF from the obtained materials, the release data were calculated according to six kinetics models as follows: zero-order kinetics (ZO),^29^ first-order kinetics (FO),^9^ second-order kinetics (SO),^30^ the Hixson–Crowell model,^30^ the parabolic model,^31,32^ and the Bhaskar model.^33^

## Results and discussion

### ACF–LDH intercalation compounds

**Fig. 3:**
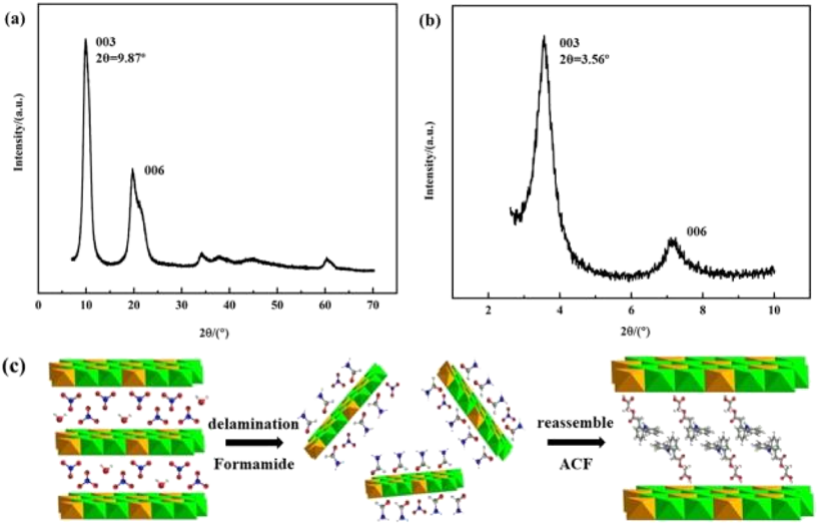
XRD patterns of (a) pristine LDH and (b) ACF–LDH. (c) Schematic illustration of the orientation of ACF–LDH.

As reported in previous literature,^28^ the system became cloudy when the LDH nanosheet suspension was dropped into the ACF–DMSO solution, indicating that the co-assembly process had occurred. Fig. 3 shows the XRD patterns of the pristine LDH and ACF–LDH nanohybrids. In comparison with the pristine LDH sample, the 003 basal reflection patterns of the co-assemblies shifted to 2*θ* = 3.56, indicating that the *d*-spacing value was 2.476 nm. The ACF molecules were successfully intercalated to the interlayer of LDHs. Given that the thickness of the brucite-like layer of LDH has been reported to be about 0.48 nm,^34^ the gallery heights of the ACF–LDH nanohybrids were assumed to be about 1.996 nm. As calculated, the length and width of the ACF molecule are 12.325 and 9.740 Å, respectively, which are smaller than the gallery heights. A possible orientation of the ACF molecules in the LDH gallery was proposed, a schematic of which is illustrated in Fig. 3. The drug molecules should be arranged in bi-layers in the interlayer and are slightly titled with the negative carboxylate groups towards the positive brucite-like layers, which is consist with the diclofenac–LDH nanohybrids.^9,35^

### FT-IR

**Fig. 4:**
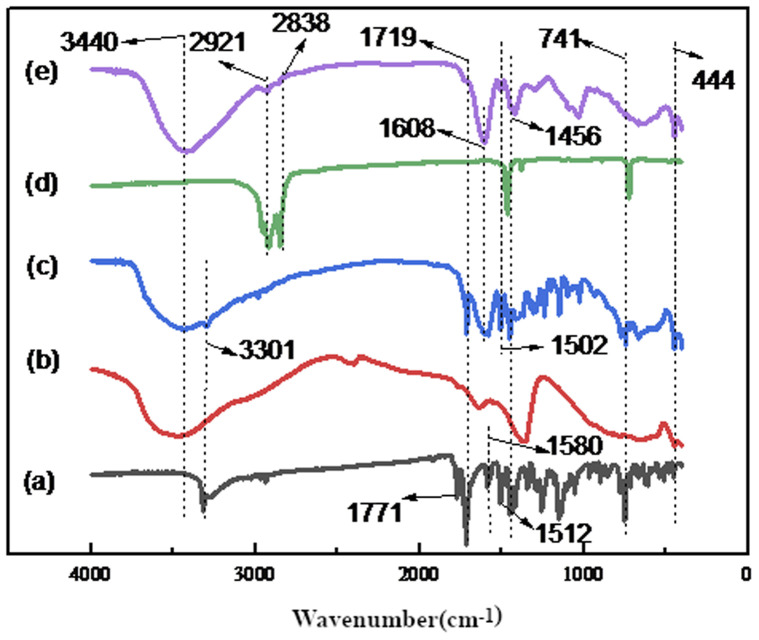
FT-IR spectra of (a) aceclofenac, (b) pristine LDH, (c) ACF–LDH, (d) sodium alginate, and (e) the ACF–LDH/Alg microspheres.

Fig. 4 shows the FT-IR spectra of the pristine LDH, the ACF–LDH and the ACF–LDH/Alg microspheres. Those of aceclofenac and sodium alginate are also shown in the same figure for comparison with the obtained samples. In the FT-IR spectra of the pristine LDH, the ACF–LDH and the ACF–LDH/Alg microspheres (Fig. 4b, c, and e), the strong and broad band centered around 3440 cm^−1^ was attributed to the stretching of the hydroxy groups, and the sharp peak at 444 cm^−1^ were assigned to metal–oxygen (M–O) lattice vibrations. For the pristine LDH, the band at 1631 cm^−1^ was derived from the deformation of water, whereas the sharp characteristic peaks at 1379 and 829 cm^−1^ were attributed to the interlayer nitrate ions. Pure aceclofenac showed sharp characteristic peaks at 3301, 1771, 1719, 1252, 1150, and 741 cm^−1^ representing the N–H, C=O, O–H, C–N, –C=O, and C–Cl groups on the phenyl groups, respectively.^36^ Characteristic bands are observed for the Alg beads at 2921 and 2838 cm^−1^ (aliphatic C–H stretching vibrations), which are also observed for the ACF–LDH/Alg microspheres. In the spectra of aceclofenac, the ACF–LDH and the ACF–LDH/Alg microspheres (Fig. 4b, c, and e), the peak at 1456 cm^−1^ was attributed to the vibration of the benzene skeleton.^35^ The peaks at 1580 and 1512 cm^−1^ were attributed to the asymmetric and symmetric stretching vibrations of the carboxyl group, while they were shifted to 1608 and 1502 cm^−1^ in the ACF–LDH nanohybrids, suggesting the existence of hydrogen bonding and electrostatic interactions between the carboxyl groups and the LDH layers, as previously reported in the literature.^28^

### SEM

**Fig. 5:**
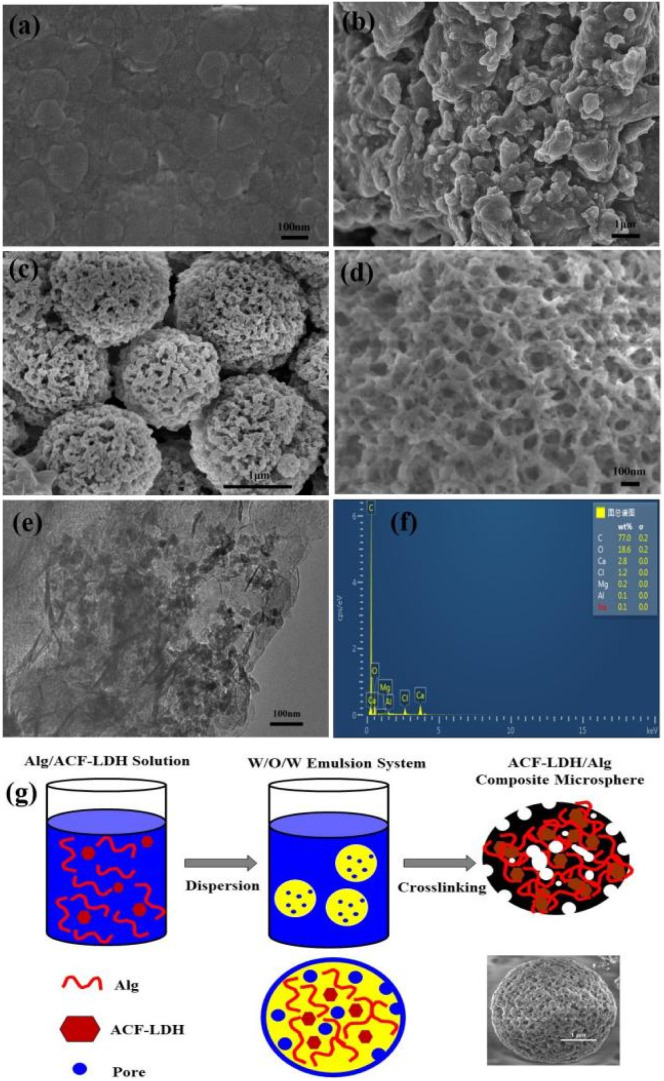
SEM images of (a) pristine LDH, (b) ACF–LDH, and (c and d) the ACF–LDH/Alg microspheres. TEM image (e) and EDS data (f) of the ACF–LDH/Alg microspheres. (g) Schematic illustration of the processes for producing the ACF–LDH/Alg composite microspheres.

Fig. 5 shows the SEM images of the pristine LDH, the ACF–LDH, and the ACF–LDH/Alg microspheres. The pristine LDH sample takes the form of common hexagonal platelets with a lateral size of about 100 nm, whereas the ACF–LDH nanohybrids showed an irregular hexagonal shape. The change of morphology may be due to the layers of LDH being partly dissolved in formamide when exfoliated to nanosheets. The ACF–LDH/Alg microspheres were successfully obtained *via* an emulsion method, as illustrated in Fig. 5c. The average size of the ACF–LDH/Alg microspheres was 2 µm. It should be noted that the ACF–LDH/Alg microspheres presented as a rough surface filled with holes. The numerous pore structures of the microspheres can be seen clearly, which are about 100 nm in diameter (shown in Fig. 5d). The ACF–LDH nanohybrids and alginate anions interacted with each other *via* electrostatic forces and cohered together to form the wall of holes, as illustrated in Fig. 5e. As shown in Fig. 5g, the ACF–LDH nanohybrids and sodium alginate mixed and interacted with each other first. With the help of the Span 80 emulsion, the ACF–LDHs/Alg were well dispersed in the paraffin. When the Ca^2+^ was diffused to the ACF–LDHs/Alg water phase, the cross-linking of alginate occurred. The key factor in the formation of the porous structure is the emulsion stabilized by the Span 80. The schematic representation of the ACF–LDHs/Alg construction is shown in Fig. 5g. EDS data also proved the presence of double layered hydroxides, calcium alginate and aceclofenac. The element distribution map indicated high Ca content, which corroborated the assumption that the Ca^2+^ replaces Na^+^ during crosslinking. The presence of Mg content and Al content revealed Mg–Al hydrotalcites in the microspheres and the Cl content revealed the aceclofenac content in the microspheres.

### TG–DSC

**Fig. 6:**
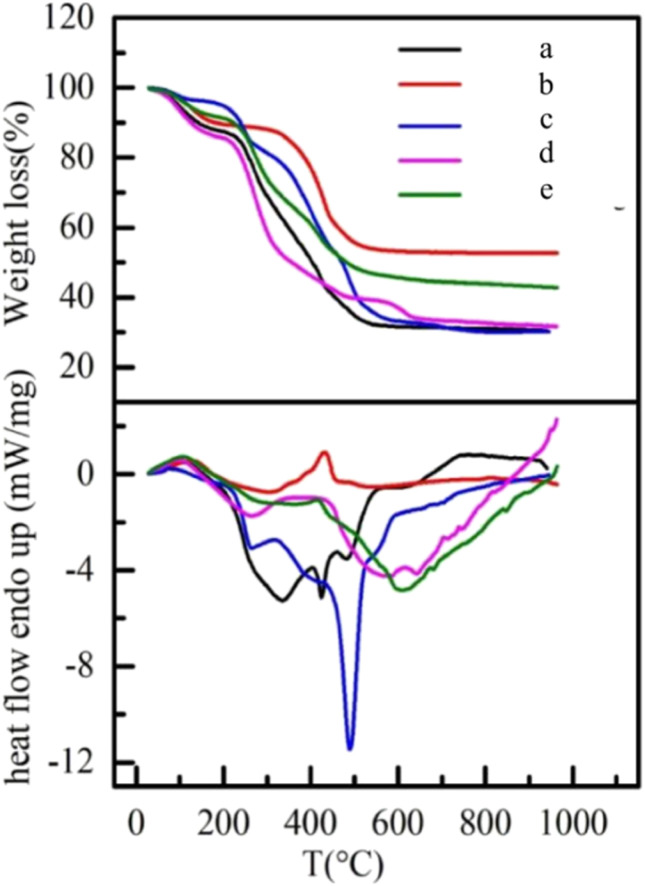
TG–DSC curves of (a) aceclofenac, (b) pristine LDH, (c) ACF–LDH, (d) the ACF–Alg microspheres and (e) the ACF–LDH/Alg microspheres.

Fig. 6 illustrates the TG–DSC curves of aceclofenac, pristine LDH, ACF–LDH, the ACF–Alg microspheres and the ACF–LDH/Alg microspheres. The first stages in these thermograms from 20 to 200 °C are attributed to the loss of free and crystalline water molecules. With an increase in the temperature, the thermo-decomposition behaviors become complicated. The remaining mass loss in pristine LDH above 350 °C is due to the dehydroxylation of the hexagonal layers (Fig. 6b).^9^ For pure aceclofenac (Fig. 6a), four main exothermic peaks at 331.5, 422.7, 483.8, and 627.1 °C accounting for a weight loss of 65% appeared in the curve due to thermal decomposition. While intercalated into the interlayer of LDH, only three exothermic peaks at 262.5, 381.0, and 489.2 °C were observed for the ACF–LDH nanohybrids. The obvious differences can be attributed to the interaction between the LDH layers and the aceclofenac anions. As reported in the literature,^9^ the drug loading in ACF–LDH can be calculated as 27% for the mass loss above 450 °C. The intercalation of ACF to form ACF–LDHs enhances the thermal stability of ACF efficiently. For the ACF–LDH/Alg microspheres, the second stage of weight loss from 220 to 385 °C with an exothermic peak at 323.0 °C is attributed to the decomposition of alginate, which occurred at 261.9 °C for the ACF/Alg microspheres. The final process of weight loss for ACF–LDH/Alg at 607.7 °C corresponds to the decomposition of the aceclofenac anions, which is higher than for ACF–LDHs. The total mass loss of the ACF–LDH/Alg microspheres is 45%, less than the loss of ACF–LDH. Overall, comparing the TG–DSC curves of ACF, ACF–LDH and the ACF–LDH/Alg microspheres, the thermal stability of the ACF molecules is greatly improved. This may be related to the intercalation of ACF and the interaction of ACF–LDH with alginate to form composites.^37^

### *In vitro* drug release

**Fig. 7:**
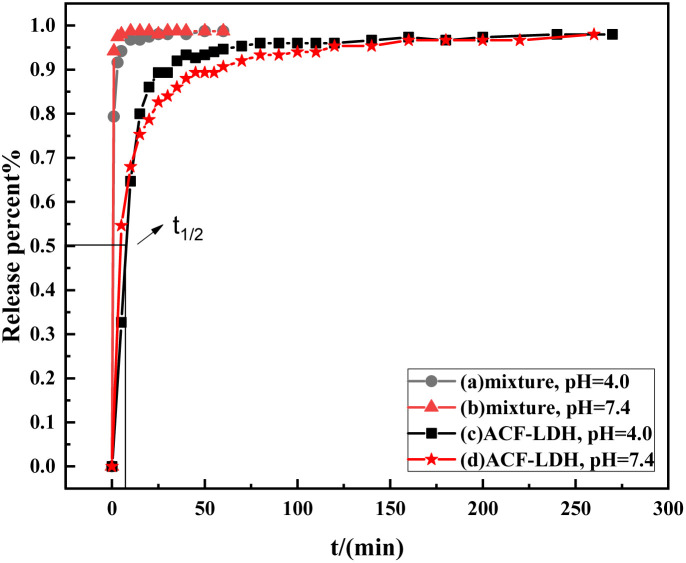
Release profiles of (a) a physical mixture, pH = 4.0, (b) a physical mixture, pH = 7.4, (c) ACF–LDH, pH = 4.0, and (d) ACF–LDH, pH = 7.4.

Fig. 7 shows the *in vitro* release profiles of ACF from the ACF–LDH nanohybrids compared with the physical mixture in PBS (pH 7.4 and 4.0) at 37 °C. The release of ACF from the physical mixture occurred quickly, and was complete within the first 10 min due to dissolution of ACF in PBS. In contrast, the ACF was released at a slower rate from the nanohybrids, with only 95% having been released in 150 min in PBS at pH 7.4. This was a little slower than the release of ACF from the ACF–LDH nanohybrids in PBS at pH 4.0. In the first 50 min, the release of ACF from the ACF–LDH nanohybrids was rapid and then the release slows down, which is similar to other nanohybrids reported in the literature.^9^

**Fig. 8:**
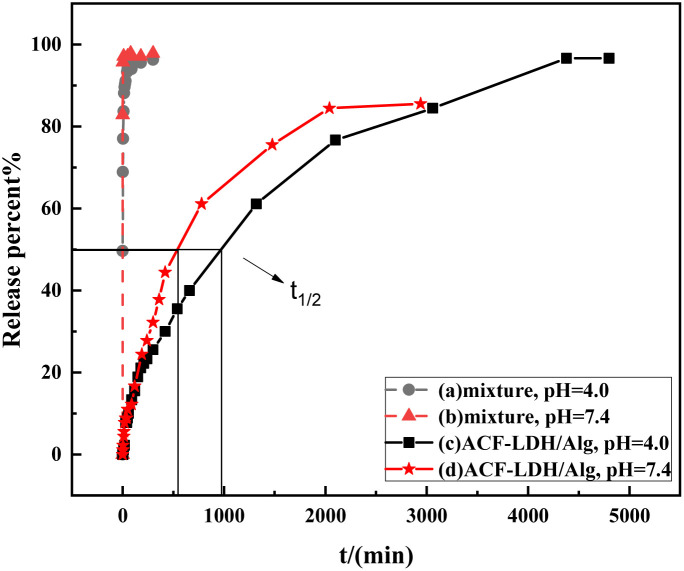
Release profiles of (a) a physical mixture, pH = 4.0, (b) a physical mixture, pH = 7.4, (c) the ACF–LDH/Alg microspheres, pH = 4.0, and (d) the ACF–LDH microspheres, pH = 7.4.

Fig. 8 illustrates the *in vitro* release profiles of ACF from the porous ACF–LDH/Alg microspheres in pH 7.4 and pH 4.0. Different from the release data of the physical mixture and the ACF–LDH nanohybrids, the release profiles of the porous ACF–LDH/Alg microspheres show a sustained and pH-responsive drug release. Notably, 50% of the incorporated drug was released within 16 h at pH 4.0, whereas 62% of the drug was released at pH 7.4. After 50 h, 75% and 74% of ACF was released from the ACF–LDH/Alg microspheres at pH 7.4 and pH 4.0, respectively. Compared with previously reported aceclofenac sustained-release systems, the obtained microspheres demonstrate unique drug release behaviors.^38–40^ This outcome is related to the intercalation of ACF into the interlayer of the LDHs and the cross-linking of alginate and Ca^2+^. Hence, porous ACF–LDH/Alg microsphere swelling was reduced in the presence of PBS because of the interaction of the LDH nanoparticles and the alginate anions, especially in under acidic conditions. Thus, release of the ACF molecules follows two procedures and the formulation exhibited sustained drug release characteristics. As the nanohybrids and microspheres represent a multi-component system, the release data were fitted to the following kinetic equations for further understanding the mechanism of the ACF release from drug delivery system. The Pearson's correlation coefficients for each of these models are shown in Table 1.

**Table 1:** The Pearson's correlation coefficients for each kinetic model

Sample	pH	Zero order	First order	Second order	Hixson–Crowell's cube-root equation	Parabolic model	Bhaskar model
ACF–LDH	4.0	0.2206	0.1514	0.9999	0.2346	0.9942	0.9308
ACF–LDH	7.4	0.4951	0.4243	0.9999	0.5105	0.9966	0.9413
ACF–LDH/Alg	4.0	0.9039	0.6800	0.9987	0.9111	0.9793	0.9935
ACF–LDH/Alg	7.4	0.8205	0.4525	0.9953	0.8291	0.9085	0.9972

It can be seen from Table 1 that for the ACF–LDH nanohybrids, the kinetics data for the release followed the second-order and parabolic kinetic models. This is an indication that the release process follows a diffusion-controlled process with strong guest–host interactions.^9^ For the porous ACF–LDH/Alg microspheres, the kinetics data for the release followed the second-order and Bhaskar kinetic models. This illustrates that the release kinetics are complicated and are driven by various factors, while the intra particle diffusion was the rate limiting step.^33^

## Conclusion

In summary, using an emulsion method, we made porous LDH/Alg microspheres loaded with aceclofenac as a pH-responsive oral drug delivery system. The ACF molecules are intercalated into the interlayers of the LDH, which were further mixed with calcium alginate to form porous LDH/Alg microspheres. ACF–LDH nanohybrids and alginate anions interacted with each other and cohered together to form the walls of pores resulting in numerous pore structures with a size of about 100 nm. The obtained porous LDH/Alg microspheres possess improved thermal stability and pH-responsive release performance for ACF molecules. Overall, the ACF–LDH/Alg microspheres may reduce stomach irritation and show promise for future use in drug delivery systems.^16,33^

## Conflicts of interest

There are no conflicts to declare.

## Supplementary Material

RA-OLF-D6RA03347D-s001

## Data Availability

All relevant data are within the paper and its supplementary information (SI). Supplementary information is available. See DOI: https://doi.org/10.1039/d6ra03347d.
