# Supplementary material for: Development and characterization of aceclofenac–LDH/alginate porous microspheres for pH-responsive drug release
Source: RSC Adv. 2026 Aug 12;16(40):47171–7. doi: 10.1039/d6ra03347d (PMC13464433; doi:10.1039/d6ra03347d)
Supplement: Supplementary file 1 [file RA-016-D6RA03347D-s001.pdf]

Supplementary Information (SI) for RSC Advances.
This journal is © The Royal Society of Chemistry 2026

Supporting Information

Development and Characterization of aceclofenac-LDH/alginate porous microspheres for pH-responsive drug release

Yanling Chen, Xiaowen Wu*

School of Resources and Environment Engineering, Shandong Agriculture and Engineering University, Jinan , 250100 , China.

Corresponding Author's e-mail: wuxiaowen1114@163.com

List of Supplementary Figures:

Figure S1. Coassembly process of LDH nanosheets and aceclofenac anions

Figure S2. XRD patterns of ACF-LDH nanohybrids for $2\theta=2\sim10°$

Figure S3. Fitting plot of the pseudo-second order kinetic model for ACF-LDH

Figure S4. Fitting plot of Parabolic kinetic model for ACF-LDH

Figure S5. Fitting plot of the pseudo-second order kinetic model for ACF-LDH/Alg microspheres

Figure S6. Fitting plot of Bhaskar kinetic model for ACF-LDH/Alg microspheres

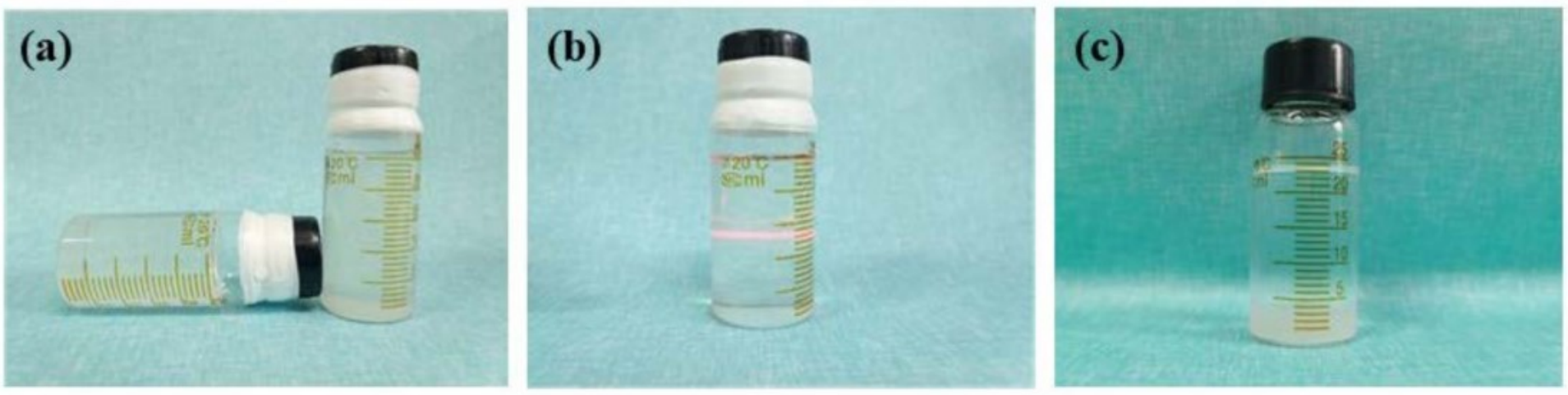

Figure S1. Coassembly process of LDH nanosheets and aceclofenac anions

Nanosheets of Mg_3Al-NO_3 LDH dispersed in formamide (a), Dyndall effect of LDH nanosheets colloid (b), cloudy suspension of ACF-LDH (c)

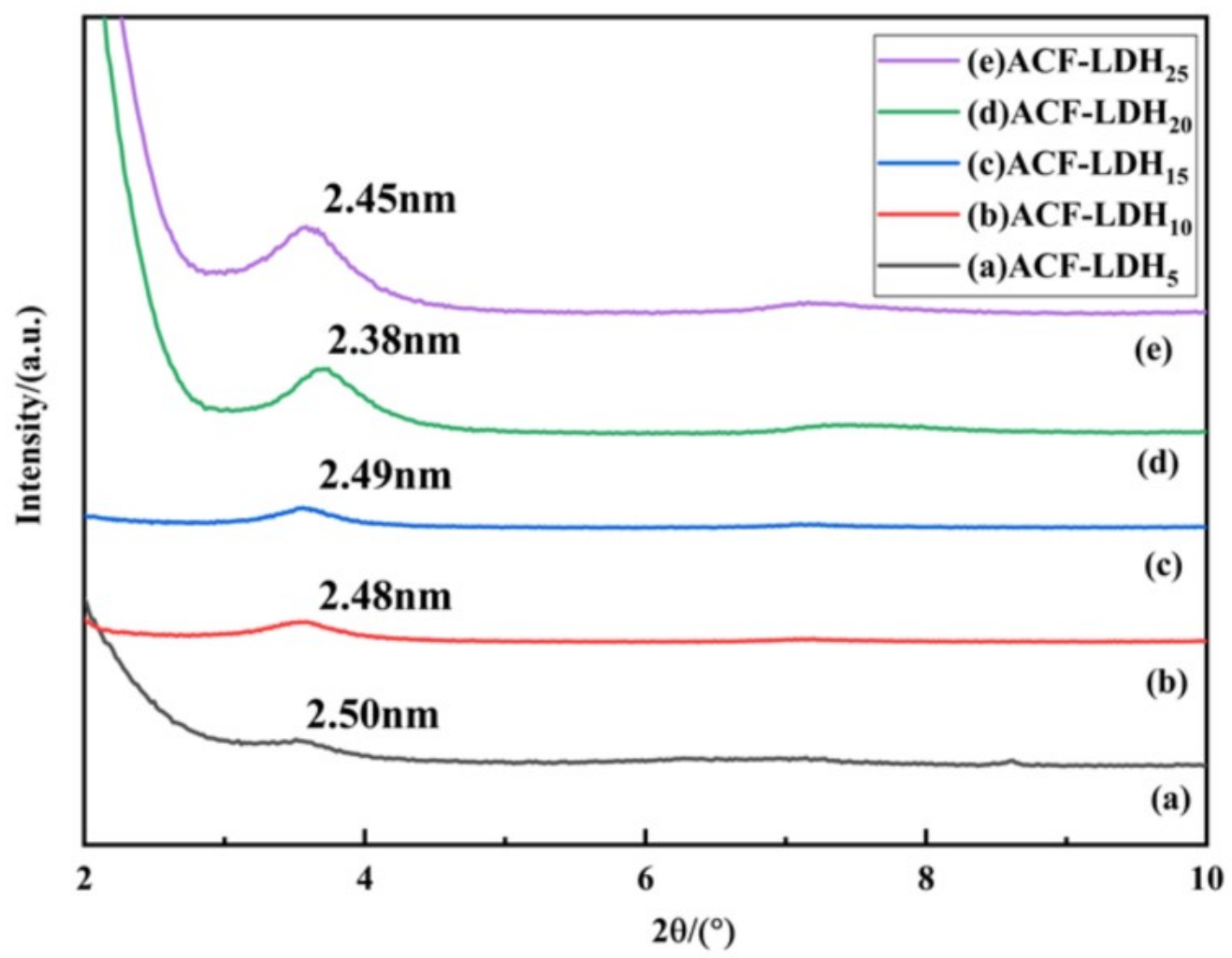

Figure S2. XRD patterns of ACF-LDH nanohybrids (different drug loading) for 2θ = 2~10°

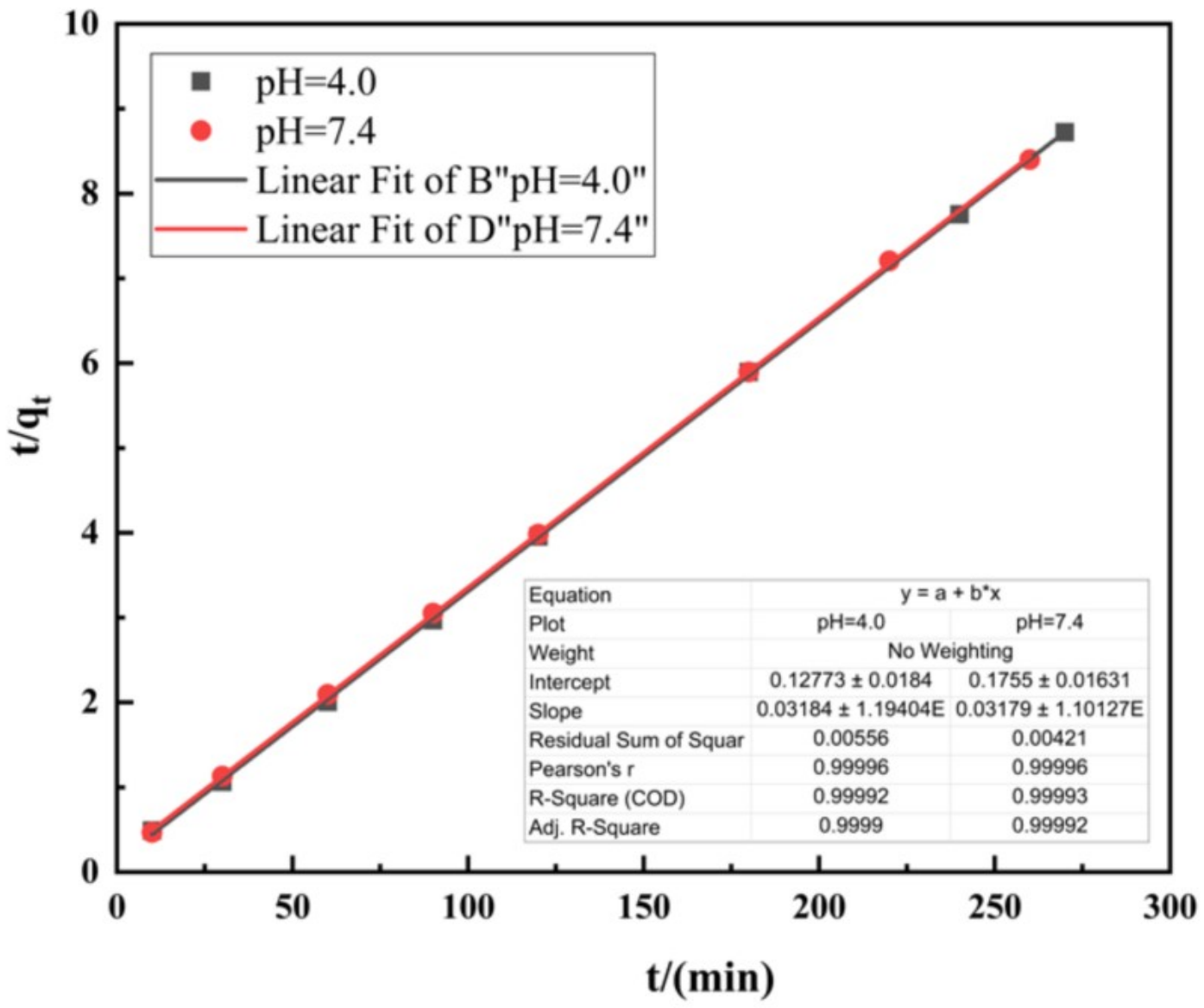

Figure S3. Fitting plot of the pseudo-second order kinetic model for ACF-LDH

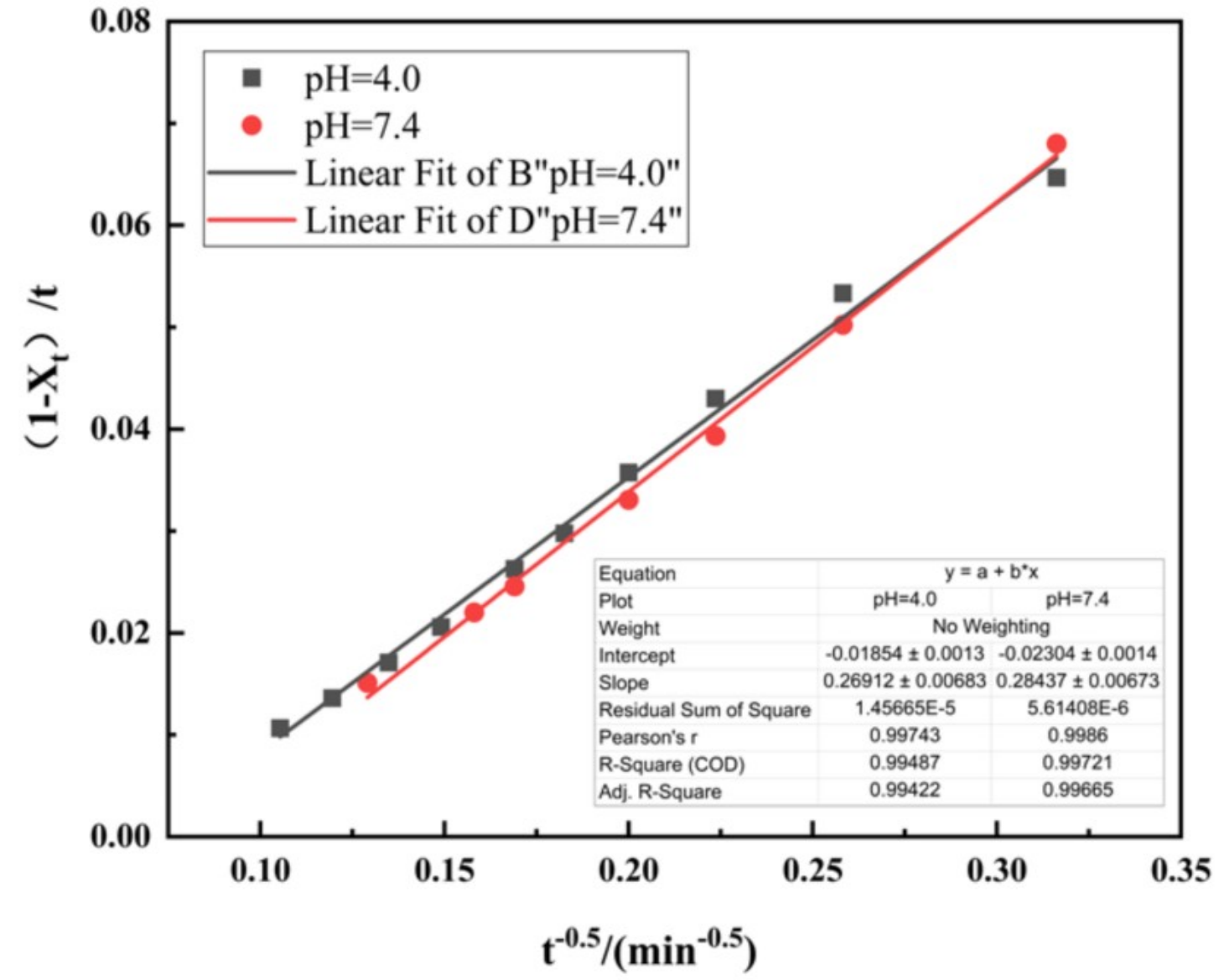

Figure S4. Fitting plot of the Parabolic kinetic model for ACF-LDH

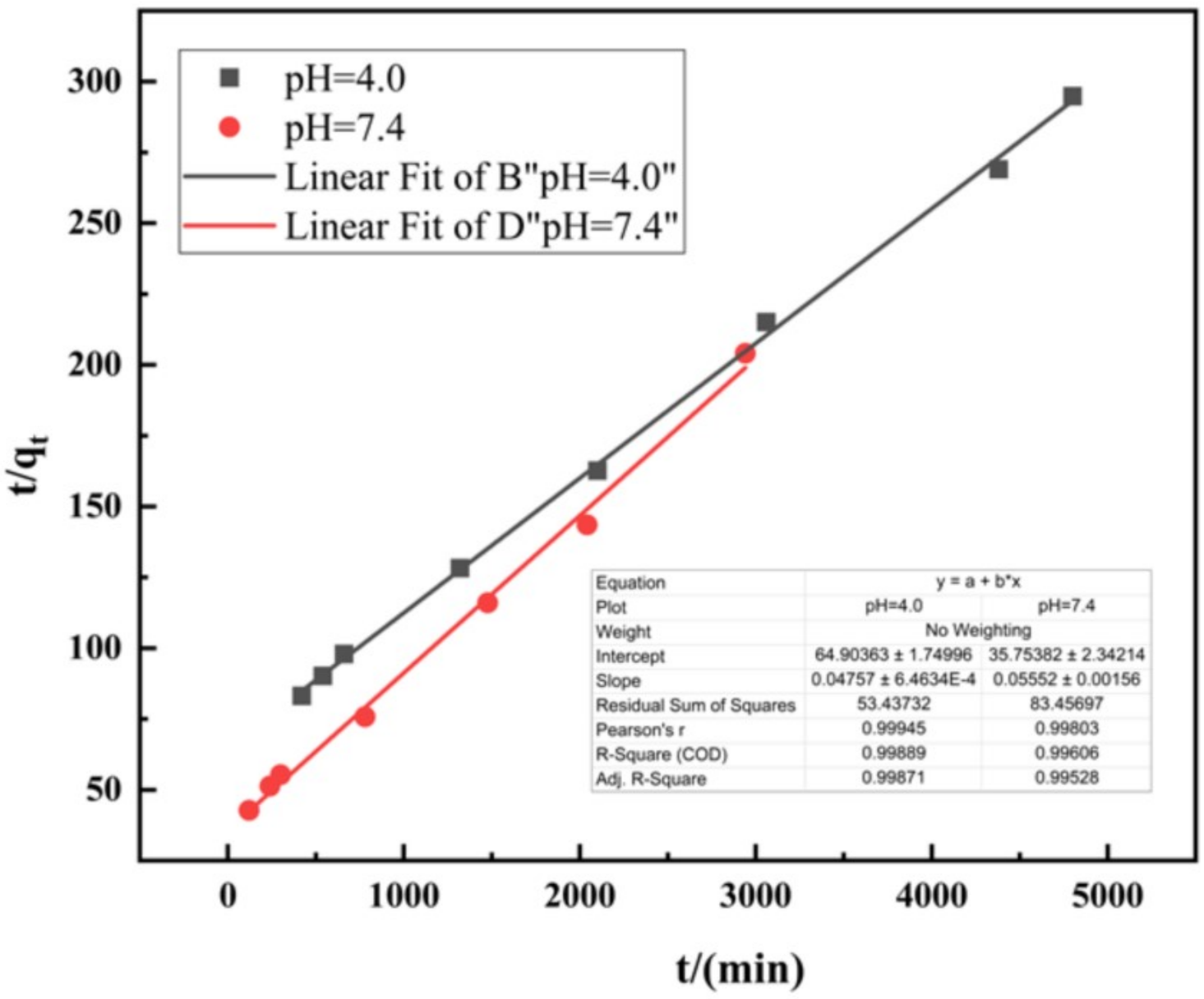

Figure S5. Fitting plot of the pseudo-second order kinetic model for ACF-LDH/Alg microspheres

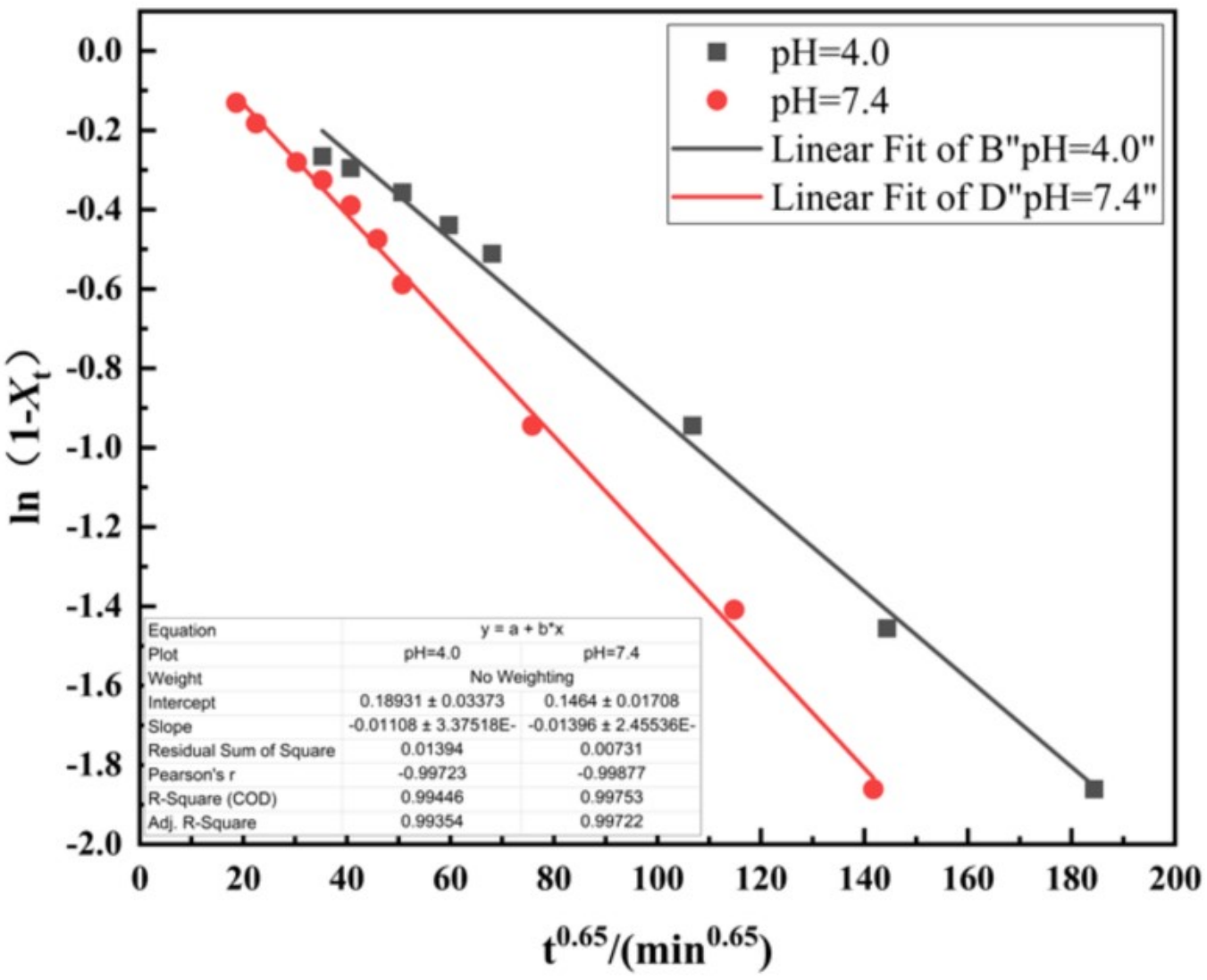

Figure S6. Fitting plot of the Bhaskar model for ACF-LDH/Alg microspheres
